# A gastric neuroendocrine carcinoma with good prognosis after chemotherapy combined with immunotherapy: A case report (CARE-compliant)

**DOI:** 10.1097/MD.0000000000036568

**Published:** 2023-12-15

**Authors:** Yuanzheng Zhao, Rong Yang, Hongxia Lu

**Affiliations:** a Department of Clinical Medicine, Fenyang College Shanxi Medical University, Shanxi, China; b Department of Gastroenterology, Shanxi Province Cancer Hospital/Shanxi Hospital Affiliated to Cancer Hospital, Chinese Academy of Medical Sciences/Cancer Hospital Affiliated to Shanxi Medical University, Shanxi, China.

**Keywords:** gastric neuroendocrine carcinoma, immunotherapy, drug resistance

## Abstract

**Introduction:**

Gastric neuroendocrine carcinoma (GNENs), as a highly malignant gastrointestinal tumor, is characterized by easy postoperative metastasis, and its prognosis has always been poor compared to other gastrointestinal tumors. Due to its rarity, there have been few case reports and studies on it. As of now, there is no clear diagnosis and treatment sequence and guidelines. In this report, we present a case of GNENs that underwent first-line treatment after surgery. The patient achieved an excellent prognosis and survival after a combination of chemotherapy resistance and immunotherapy. This report aims to provide valuable reference and guidance for the treatment of GNENs in the future. Therefore, we conducted a literature review on GNENs.

**Patient concerns:**

A 61-year-old man was admitted to the hospital with complaints of upper abdominal distension and discomfort that had been persistent for 1 month. During the endoscopy examination, a 2.5 cm irregular deep mucosal defect was observed at the center of the gastric angle. The defect appeared covered with dirty moss, had a hard texture, and exhibited a tendency to bleed upon contact.

**Diagnosis:**

Biopsy results confirmed the presence of a medium to poorly differentiated adenocarcinoma in the gastric horn. Subsequently, the patient underwent surgery, and the removed specimen was diagnosed as GNENs.

**Intervention:**

Postoperative chemotherapy combined with immunotherapy

**Outcome:**

The patient in this case achieved a good prognosis and extremely long survival [overall survival > 3 years+] after receiving first-line treatment, which included chemotherapy, drug resistance and immunotherapy, and is currently in good health condition. The tumor is not sensitive to the standard EP regimen for neuroendocrine carcinoma, but after being replaced with oxaliplatin based regimen combined with immunotherapy, partial response was obtained, indicating a synergistic effect between chemotherapy and immunity. After treatment, it remained stable for a considerable period of time.

**Conclusion:**

Immunotherapy, as a new mode of cancer treatment can provide new guidance and ideas for the treatment of GNENs.

## 1. Introduction

Neuroendocrine tumors (NETs) are widely recognized as progressive neoplasms that originate from neuroendocrine cells and are commonly found in the gastrointestinal tract, pancreas, and lungs.^[1]^ Gastrointestinal neoplasms, also known as gastroenteropancreatic NETs, account for two-thirds of all NETs.^[2]^ The incidence of gastroenteropancreatic NETs has been increasing in recent decades.^[3,4]^ GI NETs have now become the second most common gastrointestinal malignancies.^[5,6]^ In 2011, the World Health Organization classified gastric NETs into 3 categories: NETs, neuroendocrine carcinomas, and mixed glandular neuroendocrine carcinomas.^[7]^ Despite improvements in the prognosis of gastric cancer in recent years, high-grade gastric cancer is still characterized by rapid progression and chemotherapy resistance, leading to a poor prognosis.^[8]^ Cohort studies conducted by Lin et al have shown that patients with gastric neuroendocrine carcinoma (GNENs) are more prone to distant recurrence and have a worse prognosis compared to gastric adenocarcinoma or even poorly differentiated gastric adenocarcinoma.^[7]^ Additionally, research by Gaur et al has demonstrated that metastatic gastrointestinal NETs often do not respond well to chemotherapy.^[9]^ GNENs is a rare type of gastric cancer, accounting for approximately 3% of all gastrointestinal NETs and 0.3% of all gastric malignancies.^[10]^ Due to its rarity, there is currently no clear diagnosis and treatment guideline available. In this study, we present a rare case of highly malignant GNENs from Shanxi Hospital of the Chinese Academy of Medical Sciences Oncology Hospital (Shanxi Provincial Oncology Hospital), which showed significant efficacy and an extremely long survival time (OS > 3 years+) after chemotherapy combined with immunotherapy. Our diagnostic methods in this case include PE, laboratory testing, imaging [computer tomography (CT), magnetic resonance imaging (MRI), pathology], questionnaires. The aim of this report is to provide valuable reference and guidance for the treatment of GNENs in the future. The patient in this study have reached adulthood and have received and provided informed consent for the publication of the case from his brother after consultation.

## 2. Case report

A 61-year-old Chinese male was admitted to our hospital in April 2020 due to discomfort caused by upper abdominal distension for over a month.

His past medical history, family history, psychological and social history, related complications, and genetic history are all normal. Upon admission, there were no abnormalities in the physical examination, with a height of 159 cm, a weight of 42 kg, and a body surface area of 1.35 m^2^.

Blood tests did not detect increased white blood cells or anemia, and liver function tests were normal. The level of tumor markers is within the normal reference range: carcinoembryonic antigen, 1.34 ng/mL (normal value < 3); carbohydrate antigen19-9 (CA19-9), 9.76 U/mL (normal value < 37); carbohydrate Antigen72-4 (CA72-4), 3.38 U/mL (normal value < 10); carbohydrate Antigen50 (CA50), 3.18 U/mL (normal value < 20).

**Figure 1.**
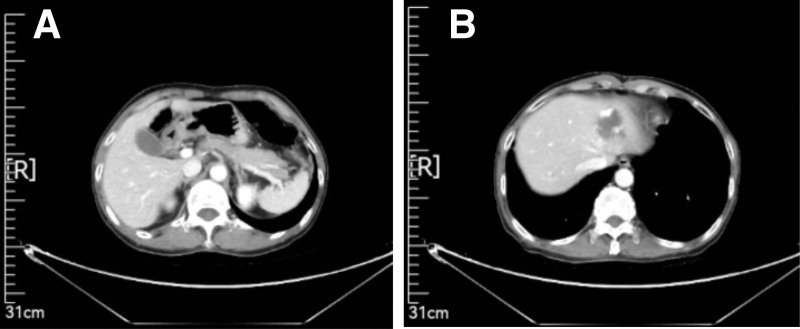
CT (April 26, 2020) revealed a slightly thickened gastric antrum and a left lobe hemangioma of the liver.

**Figure 2.**
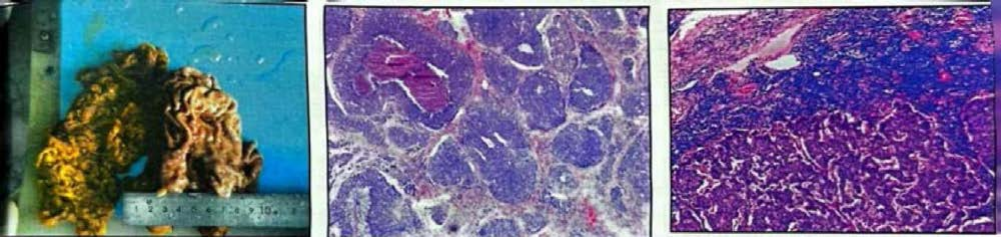
Postoperative specimen biopsy pathology revealed: Gastric angle neuroendocrine carcinoma (large cell type), focal adenoid structure, ulcerative type (Lauren type: diffuse type), tumor size 2 × 2 × 2 cm, infiltration of the gastric wall to the subserous membrane, visible tumor thrombi in the veins, no clear nerve involvement.

Endoscopic forceps were used to perform pathological biopsy of the gastric horn, indicating moderate to poorly differentiated adenocarcinoma. CT (April 26, 2020) revealed a slightly thickened gastric antrum and a left lobe hemangioma of the liver (Fig. 1). The patient met the surgical indications and underwent laparoscopic radical gastrectomy for gastric cancer under general anesthesia in our hospital on May 11, 2020. There was no ascites found in the book, and the size of the gastric antrum tumor was about 4 * 4 * 3 cm, invading the serous layer of the gastric wall. There was no lymph node enlargement in the abdominal cavity. Postoperative specimen biopsy pathology revealed: Gastric angle neuroendocrine carcinoma (large cell type), focal adenoid structure, ulcerative type (Lauren type: diffuse type), tumor size 2 × 2 × 2 cm, infiltration of the gastric wall to the subserous membrane, visible tumor thrombi in the veins, no clear nerve involvement (Fig. 2); upper and lower cutting edges and omental tissue: no cancer found; lymph node metastatic cancer and cancer nodules: 4/16 on the small curvature side, 0/6 on the large curvature side; the immunohistochemical results showed that Pan-Cytokinetin antibody (AE1/AE3) (+), Proliferation Index67 (Ki67) (approximately 70%+), cytokeratin7 (CK7) (partially+), cytokeratin20 (CK20) (−), caudal type homeobox gene-2 (CDX-2) (+), Special AT-rich sequence-binding protein2 (SATB2) (partially+), Synaptophysin (+), Chromogranin A (+), Cluster of Differentiation56 (CD56) (+), MLH1 (+), PMS2 (+), MSHS (+), Melanocyte Stimulating Hormone6 (MSH6) (+), Human epidermal growth factor receptor-2 (HER-2) (0). The patient recovered well after surgery without obvious discomfort and was discharged from the hospital.

**Figure 3.**
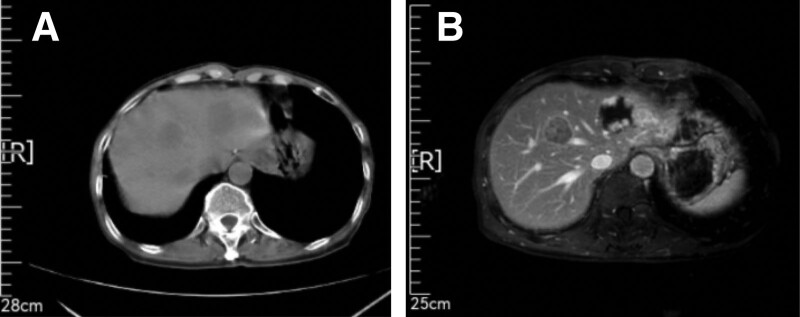
CT examination on March 16, 2021 revealed intrahepatic nodules, while MRI examination on March 16, 2021 revealed S4 segment nodules in the liver. Considering the possibility of metastasis based on medical history. Besides, multiple hemangiomas were found in the left lobe of the liver. CT = computer tomography, MRI = magnetic resonance imaging.

The patient subsequently received 6 cycles of “Etoposide + Cisplatin (EP)” chemotherapy regimen from June 12, 2020 to November 10, 2020, totaling: etoposide 100 mg ivgtt d1-d3; Cisplatin 20 mg ivgtt d1-d3; Q21d, gastrointestinal reaction level II. CT examination on March 16, 2021 revealed intrahepatic nodules, while MRI examination on March 16, 2021 revealed S4 segment nodules in the liver. Considering the possibility of metastasis based on medical history. Besides, multiple hemangiomas were found in the left lobe of the liver (Fig. 3). Reviewed and evaluated as PD (progressive disease).

**Figure 4.**
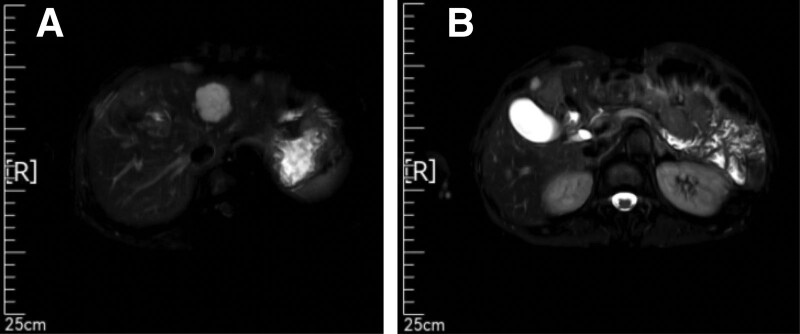
The patient MRI reexamination results on May 8, 2021 showed a mass at the junction of liver S4 and liver S8 segments, with high perfusion of the surrounding liver parenchyma, and enhanced small nodules visible in the hepatic artery phase, indicating possible metastasis. MRI = magnetic resonance imaging.

On April 19, 2021, a “color ultrasound guided microwave ablation of liver tumors” was performed under general anesthesia. Preoperative liver biopsy pathology (2021-0514): Under the microscope, atypical nuclear circular and oval cells are seen distributed in a nest shape. Combined with the medical history, metastatic cancer is considered. Combining medical history and immunohistochemical results: AE1/AE3 (+), Ki67 (approximately 70%+), CK7 (+), CK20 (−), CDX-2 (+), SATB2 (partially+), Syn (+), Chromogranin A (+), CD56 (+), Arg-1 (−), Hepatocyte (−), consistent with metastatic neuroendocrine carcinoma. The patient MRI reexamination results on May 8, 2021 showed a mass at the junction of liver S4 and liver S8 segments, with high perfusion of the surrounding liver parenchyma, and enhanced small nodules visible in the hepatic artery phase, indicating possible metastasis (Fig. 4). The evaluated effect is PD. Discharge diagnosis: postoperative gastric cancer, multiple liver metastases, T3N2M1 stage IV, microsatellite stable, Eastern Cooperative Oncology Group score of 0.

**Figure 5.**
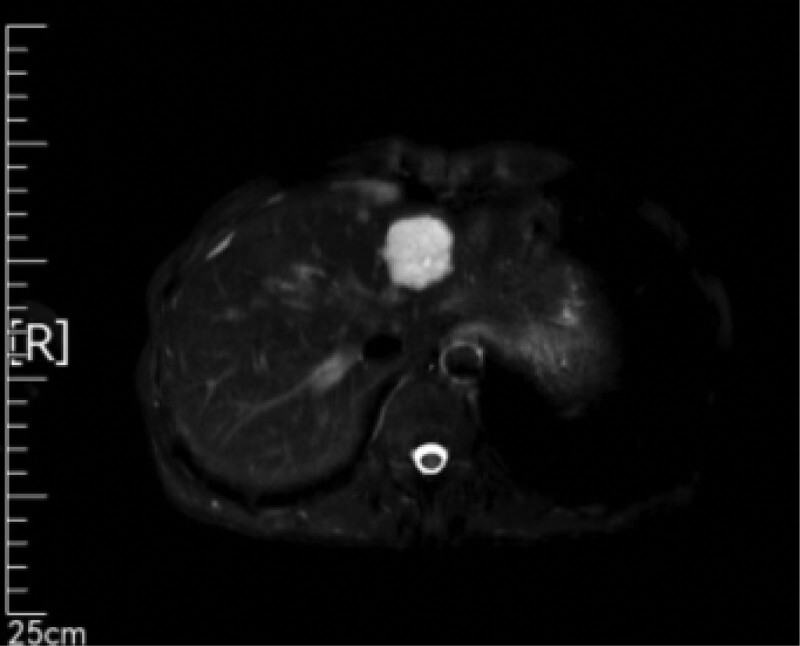
MR (2021-7-12) Indicated a reduction in lesion size at the junction of liver S4 and liver S8 segments.

**Figure 6.**
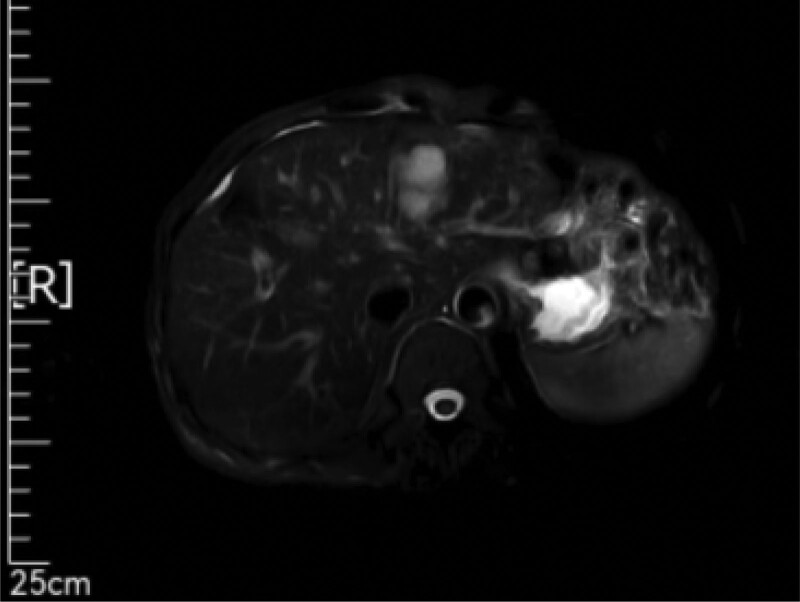
MR (2021-9-17) suggests that the lesions at the junction of liver S4 and liver S8 segments are the same as before. No small nodules were found.

From May 12, 2021 to September 18, 2021, the patient received chemotherapy combined with immunotherapy (mFOLFOX6 + Xindilimab) for 1 cycle. Due to vascular burns, the plan was changed, and the subsequent second line treatment was performed: “XELOX + Xindilimab” plan for 6 cycles (total: Xindilimab 200 mg * 7, oxaliplatin 100 mg * 1, calcium folinate 250 mg * 1, fluorouracil 3.75 g * 1, oxaliplatin 175 mg * 6, capecitabine 35 g * 6). The gastrointestinal reaction was grade II, there was no significant suppression of bone marrow hematopoietic function. The subsequent 2 follow-up examinations (July 12, 2021, September 17, 2021) were evaluated as partial response (PR) after MRI examination (Figs. 5 and 6).

**Figure 7.**
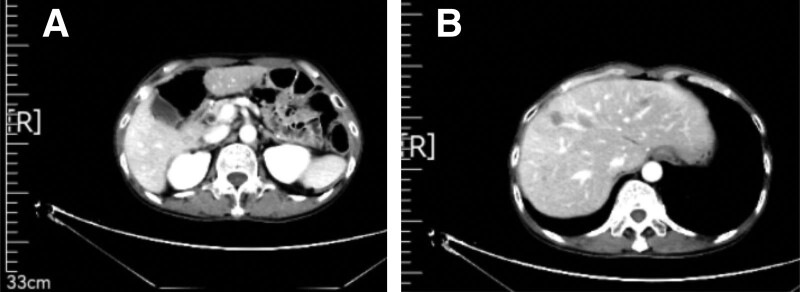
April 2, 2022, CT showed that compared to the previous CT scan of the abdomen and pelvis on December 30, 2021, there were new liver S6 and S7 segment nodules, which were considered metastatic. CT = computer tomography.

From October 25, 2021 to March 29, 2022, maintenance treatment with “Xindilimab + capecitabine” was administered for 8 cycles (total: Xindilimab 200 mg * 8, capecitabine 35 g * 8). However, during the follow-up examination on April 2, 2022, CT showed that compared to the previous CT scan of the abdomen and pelvis on December 30, 2021, there were new liver S6 and S7 segment nodules, which were considered metastatic and evaluated as PD after tumor evaluation (Fig. 7).

**Figure 8.**
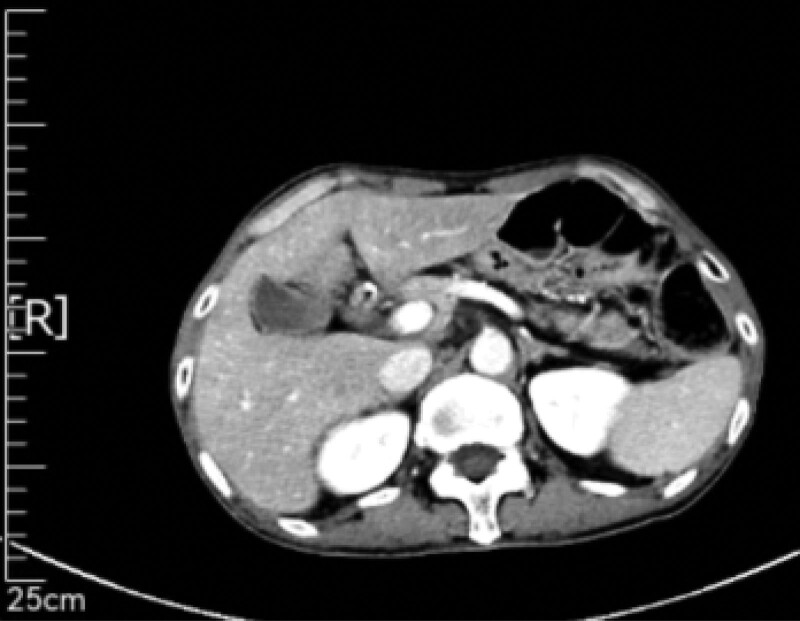
June 18, 2022, the CT examination results showed a decrease in liver S6 segment nodules compared to the old film of April 2, 2022. CT = computer tomography.

From April 19, 2022 to August 25, 2022, a third line treatment regimen was implemented: “XELOX + Xindilimab” regimen for 6 cycles, “capecitabine + Xindilimab” treatment for 1 cycle (total: Xindilimab 200 mg * 7, oxaliplatin 200 mg * 1, capecitabine 42 g * 1, oxaliplatin 175 mg * 5, and capecitabine 35 g * 5). Gastrointestinal reaction level II, bone marrow hematopoietic function level II is inhibited. During the period of June 18, 2022, the CT examination results showed a decrease in liver S6 segment nodules compared to the old film of April 2, 2022, and the tumor was evaluated as PR (Fig. 8).

**Figure 9.**
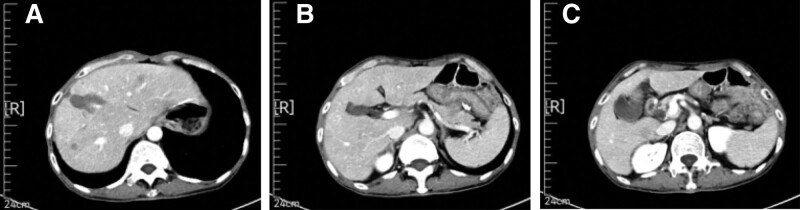
CT (2022-9-14) shows larger nodules in the S6 and S7 segments of the liver compared to previous CT images on June 18, 2022, and nodules in the anterior end of the pancreatic tail. Metastasis is considered. CT = computer tomography.

**Figure 10.**
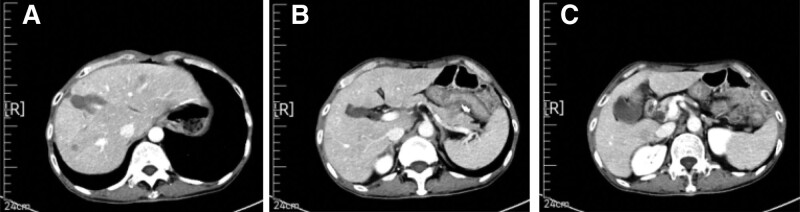
MR (2022-9-15) showed an increase in nodules in the right lobe of the liver compared to previous MR images on June 18, 2022; pancreatic tail nodule, considering metastasis.

In the CT and MRI imaging examinations on September 14, 2022, nearly 1 month after the end of the third line treatment. Compared to the old CT images on June 18, 2022, the nodules in the S6 and S7 segments of the liver were larger than before, and the nodules in the anterior end of the pancreatic tail were considered for metastasis. Compared to the old MRI film from June 18, 2022, the nodule in the right lobe of the liver is enlarged; pancreatic tail nodule, considering metastasis. The tumor was evaluated as PD (Figs. 9 and 10).

**Figure 11.**
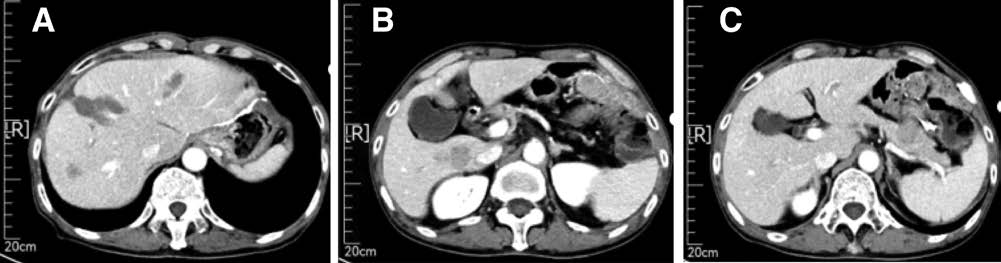
CT (2023-1-3): Compared with CT on September 14, 2022, nodules in the S7 and S6 segments of the liver increased; enlarged nodules at the junction of the pancreatic body and tail; there is a small amount of fluid accumulation around the liver, and there is no significant change in the rest. CT = computer tomography.

**Figure 12.**
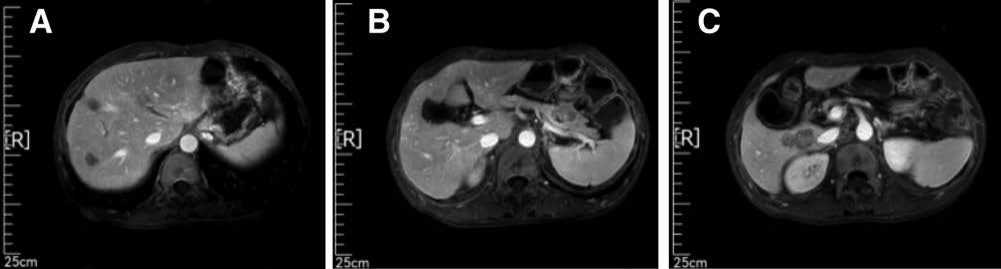
MR (2023-1-3): Compared to the old MR film on September 15, 2022, 2 metastatic nodules in the right lobe of the liver were enlarged; enlargement of metastatic nodules in the tail of the pancreas; there is a slight increase in abdominal fluid accumulation.

Subsequently, the patient underwent fourth line treatment from September 16, 2022 to December 30, 2022, using the “sofantinib + sindirizumab” regimen (total: sindirizumab 200 mg ivgtt d1 q21d, sofantinib 150 mg po qd), and there were no significant adverse reactions during the treatment period. However, subsequent imaging examinations revealed partial progression of the condition. CT (2023-13): Compared with CT on September 14, 2022, nodules in the S7 and S6 segments of the liver increased; enlarged nodules at the junction of the pancreatic body and tail; there is a small amount of fluid accumulation around the liver, and there is no significant change in the rest; MR (2023 January 3): compared to the old MR film on September 15, 2022, 2 metastatic nodules in the right lobe of the liver were enlarged; enlargement of metastatic nodules in the tail of the pancreas; there is a slight increase in abdominal fluid accumulation (Figs. 11 and 12). And after tumor evaluation, it was rated as PD.

**Figure 13.**
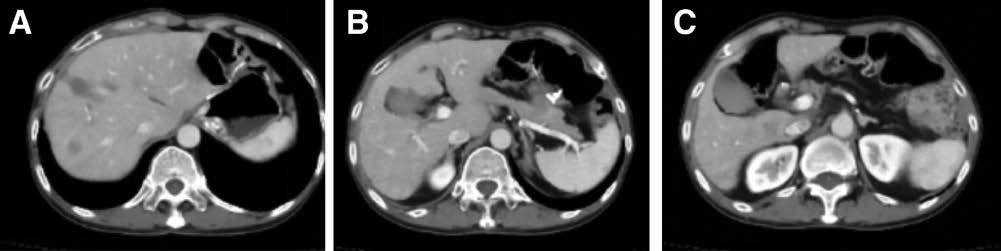
Subsequent CT examination (April 7, 2023) showed that compared to the old film, the nodules in the S7 and S6 segments of the liver were reduced; reduction of nodules at the junction of the pancreatic body and tail; there is a decrease in perihepatic fluid accumulation and a small amount of pelvic fluid accumulation, with no significant changes observed. CT = computer tomography.

From February 1, 2023 to March 15, 2023, patients underwent 3 cycles of treatment with the “Albumin Paclitaxel + Sofantinib + Sinilimab” regimen (total: Albumin Paclitaxel 300 mg ivgtt d1 q21d; Sinilimab 200 mg ivgtt d1 q21d; Sofantinib 150 mg po qd), during which a decrease in granulocyte grade III was observed. The treatment method is a fifth line treatment. Subsequent CT examination (April 7, 2023) showed that compared to the old film, the nodules in the S7 and S6 segments of the liver were reduced; reduction of nodules at the junction of the pancreatic body and tail; there is a decrease in perihepatic fluid accumulation and a small amount of pelvic fluid accumulation, with no significant changes observed (Fig. 13).

The treatment effect was good, and then continued to maintain fifth line treatment on April 10, 2023, May 5, 2023, and May 31, 2023: the “Albumin Paclitaxel + Sofantinib + Sinilimab” regimen (total: Albumin Paclitaxel 300 mg ivgtt d1 q21d; Sinilimab 200 mg ivgtt d1 q21d; Sofantinib 150 mg po qd), and the patient developed grade IV granulocyte reduction, which was subsequently prevented by PEG-rhG-CSF.

## 3. Discussion

After receiving first-line treatment including chemotherapy, drug resistance, and immunotherapy without any clear guidelines to follow, this patient achieved a good prognosis and an extended survival period (OS > 3 years, still in good condition to this day). The tumor is not sensitive to the standard EP regimen for neuroendocrine carcinoma, but after replacing with oxaliplatin based regimen combined with immunotherapy, PR was obtained, indicating a synergistic effect between chemotherapy and immunity. And after maintenance treatment, it remains stable for a considerable period of time. Previous studies have shown that intraoperative and postoperative adjuvant chemotherapy for gastrointestinal tumors directly affects the prognosis and survival of patients.^[7,11]^ However, due to the high malignancy and chemotherapy resistance of GNENs, the effectiveness of postoperative adjuvant chemotherapy is not satisfactory. Additionally, the prognosis of patients with GNENs compared to other gastrointestinal tumors is unclear due to its rarity.^[7]^ Immunotherapy, which has been a major advancement in clinical oncology, has successfully treated many types of cancer over the past decade.^[12]^ Recent data suggest that immunotherapy has emerged as a new paradigm for cancer treatment.^[13]^ It is currently the first-line treatment for many malignancies.^[14,15]^ However, further exploration is needed to determine whether it can be used as a standard treatment method for rare tumor diseases like GNENs. Therefore, our case report can provide a new approach and support for postoperative chemotherapy combined with immunotherapy for gastric NETs.

## 4. Introspection

The advantages of this case: In the absence of guidelines to follow, a combination of chemotherapy and immunization was performed on neuroendocrine cancer patients with poor biological behavior, and achieved OS for over 3 years; clinical benefits have still been observed in cross line immunotherapy, providing a basis for the selection of post line immunotherapy for gastric cancer in the future; the combination of systemic and local treatment is very reasonable, and every treatment decision is made under the guidance of MDT; the patient has good treatment compliance and complete imaging and pathological data.

The limitation of this case report: There are no clear guidelines and basis before treatment; due to the patient receiving the complimentary medication, which can be used for 2 years without any adverse reactions to immunotherapy, the immune medication was not timely changed after the condition progressed. There is no suitable biomarker to predict the efficacy of immunotherapy for NEC. After treatment, the patient has worsened bone marrow suppression and should consider reducing the dosage of the medication.

## Author contributions

**Data curation:** Rong Yang.

**Formal analysis:** Hongxia Lu.

**Funding acquisition:** Hongxia Lu.

**Investigation:** Rong Yang.

**Methodology:** Hongxia Lu.

**Project administration:** Hongxia Lu.

**Resources:** Hongxia Lu.

**Software:** Yuanzheng Zhao.

**Supervision:** Hongxia Lu.

**Visualization:** Yuanzheng Zhao.

**Writing – original draft:** Yuanzheng Zhao.

**Writing – review & editing:** Yuanzheng Zhao.
